# Supplementary material for: From sequence to enzyme mechanism using multi-label machine learning
Source: BMC Bioinformatics. 2014 May 19;15:150. doi: 10.1186/1471-2105-15-150 (PMC4229970; doi:10.1186/1471-2105-15-150)
Supplement: Additional file 2 — Java code of ml2db. Additional file ml2db_code.tar.gz contains the Java source code to run the multi-label machine learning experiments and save the results to database. The code’s Javadoc is included. [file 1471-2105-15-150-S2.zip › additional file 2/ml2db/ml2db/doc/mulan/core/ArgumentNullException.html]

ArgumentNullException


JavaScript is disabled on your browser.


- Overview
- Package
- Class
- Use
- Tree
- Deprecated
- Index
- Help

- Prev Class
- Next Class

- Frames
- No Frames

- All Classes

- Summary:
- Nested |
- Field |
- Constr |
- Method

- Detail:
- Field |
- Constr |
- Method


mulan.core

## Class ArgumentNullException

- java.lang.Object
- - java.lang.Throwable
  - - java.lang.Exception
    - - java.lang.RuntimeException
      - - mulan.core.MulanRuntimeException
        - - mulan.core.ArgumentNullException

- All Implemented Interfaces:
  :   java.io.Serializable

  ---

    

  ```
  public class ArgumentNullException
  extends MulanRuntimeException
  ```

  This exception is raised when a null reference is passed to a method that does not accept
  a null reference for an argument.

  Author:
  :   Jozef Vilcek

  See Also:
  :   Serialized Form

- - ### Constructor Summary

    Constructors

    Constructor and Description

    `ArgumentNullException(java.lang.String paramName)`

    Creates a new instance of `ArgumentNullException` for specified parameter.

    `ArgumentNullException(java.lang.String paramName,
    java.lang.String message)`

    Creates a new instance of `ArgumentNullException` with detailed message
    for specified parameter.
  - ### Method Summary

    Methods

    | Modifier and Type | Method and Description |
    | --- | --- |
    | `java.lang.String` | `getMessage()` |

    - ### Methods inherited from class java.lang.Throwable

      `addSuppressed, fillInStackTrace, getCause, getLocalizedMessage, getStackTrace, getSuppressed, initCause, printStackTrace, printStackTrace, printStackTrace, setStackTrace, toString`
    - ### Methods inherited from class java.lang.Object

      `equals, getClass, hashCode, notify, notifyAll, wait, wait, wait`

- - ### Constructor Detail


    - #### ArgumentNullException

      ```
      public ArgumentNullException(java.lang.String paramName)
      ```

      Creates a new instance of `ArgumentNullException` for specified parameter.

      Parameters:
      :   `paramName` - the name of the parameter which caused `ArgumentNullException`.


    - #### ArgumentNullException

      ```
      public ArgumentNullException(java.lang.String paramName,
                           java.lang.String message)
      ```

      Creates a new instance of `ArgumentNullException` with detailed message
      for specified parameter.

      Parameters:
      :   `message` - the detailed message.
      :   `paramName` - the name of the parameter which caused `ArgumentNullException`.
  - ### Method Detail


    - #### getMessage

      ```
      public java.lang.String getMessage()
      ```

      **Overrides:**
      :   `getMessage` in class `java.lang.Throwable`


- Overview
- Package
- Class
- Use
- Tree
- Deprecated
- Index
- Help

- Prev Class
- Next Class

- Frames
- No Frames

- All Classes

- Summary:
- Nested |
- Field |
- Constr |
- Method

- Detail:
- Field |
- Constr |
- Method
